# Mesenchymal stem cells derived from adipose tissue accelerate the progression of colon cancer by inducing a MTCAF phenotype *via* ICAM1/STAT3/AKT axis

**DOI:** 10.3389/fonc.2022.837781

**Published:** 2022-08-09

**Authors:** Chunling Xue, Yang Gao, Zhao Sun, Xuechun Li, Mingjia Zhang, Ying Yang, Qin Han, Chunmei Bai, Robert Chunhua Zhao

**Affiliations:** ^1^ Beijing Key Laboratory, Institute of Basic Medical Sciences Chinese Academy of Medical Sciences, School of Basic Medicine Peking Union Medical College, Center of Excellence in Tissue Engineering Chinese Academy of Medical Sciences, Peking Union Medical College Hospital, Beijing, China; ^2^ Department of oncology, Peking Union Medical College Hospital, Chinese Academy of Medical Science and Peking Union Medical College, Beijing, China

**Keywords:** ICAM-1, progression, survival, cell trafficking, AKT and STAT3 signaling

## Abstract

Previous studies have shown that the risk of colon cancer is greatly increased in people with obesity, and fat content in colorectal cancer tissue is increased in people with obesity. As an important part of tumor microenvironment, adipose-derived mesenchymal stem cells (MSCs) are also another important source of cancer-associated fibroblasts (CAFs), which may be one of the important mechanisms of affecting tumor progression. However, the mechanism is poorly defined. In the present study, CAFs were transformed from MSCs [MSC-transformed CAFs (MTCAFs)] by co-culturing with HCT116 cells. Bioinformatics and Western blotting analysis indicated a positive correlation between intercellular adhesion molecule-1(ICAM-1) and the progression of colon cancer. In clinical colon cancer specimens, we found that ICAM-1 was highly expressed and related to shorter disease-free survival, which might act as an indication for the progression of clinical colon cancer. Our data showed that ICAM-1 secreted from MTCAFs could positively promote the proliferation, migration, and invasion of colon cancer cells by activating signal transducer and activator of transcription 3 (STAT3) and Serine/threonine-protein kinase (AKT) signaling and that blocking ICAM-1 in MTCAFs reversed these effects. We further verified that ICAM-1 secreted from MTCAFs promoted tumor progression *in vivo*. Taken together, ICAM-1 plays a critical role in regulating tumor growth and metastasis, which could be a potential therapeutic target in colon cancer.

## Graphical Abstract

Parsimony diagram for the progression of cancer by MTCAF-derived ICAM1. ICAM-1 secreted from MTCAFs enhances the migration and invasion ability of colorectal cancer cells by activating the AKT and STAT3 pathway in cancer cells.

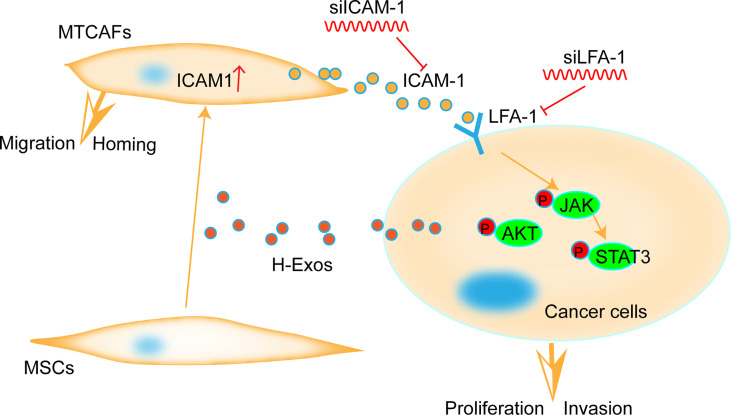

## Introduction

Mesenchymal stem cells (MSCs) are an important component of the tumor microenvironment (TME). MSCs are also referred to as “mesenchymal stromal cells”, which implies that MSCs have characteristics associated with stem cells. Importantly, MSCs are a population of adult multipotent cells that have the capacity of self-renewal and can differentiate into osteoblasts, chondrocytes, and adipocytes (1, 2). In addition, MSCs can be obtained from different tissues including the bone marrow, adipose tissues, placenta, or umbilical cord (1, 3). Several studies have demonstrated that MSCs possess multilineage differentiation potential (4) and can differentiate into cancer-associated fibroblasts (CAFs) *via* co-culturing with cancer cells that can secrete cytokines, growth factors, and CAF-specific proteins (5, 6).

Colorectal cancer is one of the most common malignancies globally, with about 1.2 million new cases and 600,000 deaths per year, accounting for the third highest incidence and the fourth leading cancer-related morbidity (7, 8). Recent studies have shown that cancer progression and metastasis are not only associated with the properties of tumor cells but also depend on the TME (9). The stroma of colon cancer forms a complex ecosystem containing immune cells, endothelial cells, and CAFs, with the latter characterized by overexpression of marker proteins, including alpha–smooth muscle actin (α-SMA) and fibroblast-activated protein (FAP) (10, 11); these provide a niche for cancer cells to modulate tumor invasion and growth (12, 13). Recent studies show that CAFs are actively involved in tumorigenesis, and it can be anticipated that the molecular characteristics of CAFs have an impact on the clinical behavior of a tumor (14–16).

Intercellular adhesion molecule-1 (ICAM-1) is a 90-kDa cell surface glycoprotein of the immunoglobulin superfamily, which has been shown to be responsible for cancer metastasis (17, 18). ICAM-1 is the most important ligand of leukocyte function–associated antigen-1 (LFA-1), which is an αLβ2 chain integrin expressed on the surface of endothelial cells and modulates the behavior of leukocytes by mediating their adhesion to other cells through its interaction with cell-surface ligands (19). In addition, the interaction between LFA-1 and ICAM-1 is involved in inflammatory responses, inflammatory pathologies, autoimmune diseases, and many cancer processes (19). ICAM-1 expression is positively related with the activation of IL-6/AKT/STAT3/NF-κB signaling pathways (20). However, the effect of knocking down ICAM-1 on tumorigenesis is unknown. STAT3 is a well-known and significant mediator of malignant progression in colorectal cancer, which is mainly activated by IL-6 (21). IL-6 binds to soluble or membrane-bound IL-6 receptor (IL-6Rα) polypeptides, which stimulates the activation of Janus kinases (JAKs), and the downstream effectors, STAT3, Shp-2-Ras, and phosphatidylinositol 3′ kinase (PI3K)–Akt (22, 23). CAFs within the TME actively contribute to sustained STAT3 activation in colorectal cancer (21). In addition, activation of IL-6-STAT3 signaling contributes fibroblasts to their conversion into CAFs in normal gastric fibroblasts (21, 24), and IL-6 enhances the proliferation of human colon carcinoma cells *in vitro* (25, 26).

Here, we sought to better understand the mechanism by which CAFs promote cell migration and invasion in colorectal cancer so as to implicate it as a potential target that could be explored further for its clinical relevance in the treatment of colorectal cancer.

## Materials and methods

### Cell culture

HCT116 cells were obtained from the Cell Resource Center, Peking Union Medical College (which is the headquarters of the National Infrastructure of Cell Line Resource, NSTI), which were cultured in Dulbecco’s Modified Eagle Medium (DMEM)/high glucose (11965092, Gibco, USA) supplemented with 10% fetal bovine serum (FBS; 16140071, Gibco, USA) and penicillin (100 IU)/streptomycin (100 µg/ml) at 37°C in a 5% CO_2_ incubator. Cells are available within 15 generations. The extraction and culture methods of MSC refer to previous studies.

### Isolation and culture of human adipose-derived MSCs

We collected adult fat samples from plastic surgery hospitals after obtaining informed consent from the donors. Using D-Hanks’ buffer, the adipose tissue was washed twice with two antibiotics (penicillin and streptomycin) and centrifuged at 800*g* for 3 min. The upper layer was transferred to a new 50-ml centrifuge tube. Then, 0.2% collagenase P (Life Technologies Corporation) was added to the tubes containing the pelleted tissue for enzymatic digestion followed by incubation at 37°C for 30 min. Subsequently, the digested adipose tissue was filtered with a 100-µm cell strainer. The sample was centrifuged at 1,500*g* for 10 min. Next, 2 × 10^6^ cells were seeded in T75 flasks and incubated at 37°C and 5% CO_2_ in a cell incubator.

### Extraction of exosomes secreted by HCT116 cells

DMEM (Life Technologies Corporation) was replaced with human adipose-derived MSC (hAD-MSC) culture medium without FBS, 36−48 h before exosome extraction. Supernatants were harvested after culture and centrifuged at 3,000*g* for 10 min to remove dead cells and cell debris. The sample was transferred to the ultrafiltration apparatus (Life Technologies Corporation) with a 100,000-kDa–molecular weight ultrafiltration membrane. Exosomes were resuspended in D-Hanks’ buffer, and the suspension was filtered with a 0.2-µm microporous membrane filter, dispensed in 1.5-ml sterile microcentrifuge tubes, and preserved at −80°C.

### Identification of exosomes using transmission electron microscopy

The purified exosomes were diluted and dropped onto a copper mesh for 5 min for precipitation. Then, filter paper was used to absorb excess liquid, and the sample was air dried. Subsequently, 3% phosphotungstic acid in water was used to counterstain the sample for 2 min. Finally, exosomes were observed using a transmission electron microscope (Olympus, Japan) and photographed.

### Exosome uptake

1,1-Dioctadecyl-3,3,3,3-tetramethylindotricarbocyaine iodide (DiR; 1 µM) (Life Technologies Corporation) is a lipophilic carbon cyanine dye that can bind lipoproteins in a manner similar to phospholipids and is embedded in the membrane of the biomass and oriented within the membrane. Diffusion movement can be used to observe cell-bound or endocytic lipoproteins under a fluorescence microscope, and this allows for semi-quantitative analysis (27). Purified exosomes were exposed to 1 µM DiR for 10 min. After incubating with MSCs for 10 h, the cells were washed with PBS three times, and the nuclei were stained with Hoechst 33342 (10 µg/ml) for 15 min at room temperature and washed with Phosphate Buffered Saline (PBS) three times. The cells were observed under a fluorescence microscope (OLYMPUS) and photographed.

### Cell–cell co-culture

A Transwell^®^ chamber (0.4 µm) (Corning) was used to co-culture the HCT116 cells with the hAD-MSCs at 1:1 ratio. The cells were passaged when cell density is 90%. Cell–cell co-culturing samples were collected at days 0, 3, 5, 7, and 9.

### siRNAs infection

Small interfering RNA (siRNAs) were used to knockdown ICAM-1 mRNA and synthesized by GenePharma company (China). The sequence of knocking down ICAM-1 is GGCTGGAGCTGTTTGAGAACA. Specific operation of virus infection was described as previous report (28).

### Western blotting analysis

Proteins were extracted from cells using IP lysis buffer (87787, Thermo Fisher Scientific) with a cocktail (4693116001, Roche, Basel, Switzerland) and PhosSTOP (4906845001, Roche). The proteins were denatured in SDS (Sigma-Aldrich) with loading buffer and boiled for 10 min at 100°C. Sodium dodecyl sulfate polyacrylamide gel electrophoresis (SDS-PAGE) was used to separate the proteins followed by the transfer of protein bands onto polyvinylidene fluoride (PVDF) membranes (Merck Millipore, Billerica, MA, USA). The membranes were then blocked with 5% milk in Tris-buffered saline–Tween 20 followed by overnight incubation at 4°C with primary antibodies. They were then washed and incubated with appropriate secondary antibodies for 1 h at room temperature, and bands were visualized using the enhanced chemiluminescence detection kit Life Technologies Corporation. The ICAM-1 (5915, 1:1,000), IL-6 (12912, 1:1,000), AKT (9272,1:1,000), Extracellularr regulated protein kinases (ERK) (4695, 1:1,000), p-ERK1/2 (4370, 1:1,000), p-JNK (9251, 1:1,000), anti-rabbit Horseradish Peroxidase labeled Anti-mouse IgG (IgG-HRP) (14708,1:2,000), Phospho-Stat3 (Tyr705, 9145, 1:1,000), Stat3 (D3Z2G, 12640, 1:1,000), Jak2 (D2E12, 3230, 1:1,000), Phospho-Jak2 (Tyr1007, 3771, 1:1,000), and anti-mouse IgG-HRP (14709, 1:2,000) antibodies were obtained from Cell Signaling Technology (Danvers, MA, USA); IL-8 (500-M08, 1:1,000) and p-Akt (ser473, 66444-1-IG, 1:2,000) antibodies were purchased from ProteinTech (Chicago, IL, USA).

### Real-time quantitative polymerase chain reaction

RNA was extracted from cell samples using TRIzol (Thermo Fisher Scientific). RNA was thawed in 30 μl of RNA free water (Applygen) and reverse-transcribed (60 µl) according to the protocol recommended for the TaKaRa M-MLV reverse transcriptase (Takara). Amplification of the gene fragment was performed. To amplify the ICMA1, IL-6, IL-8, and Glyceraldehyde-3-phosphate dehydrogenase (GAPDH) genes, one-step real-time quantitative polymerase chain reaction (RT-PCR) was performed as follows: 95°C for 5 min, 95°C for 10 s, 60°C for 40 s, 40 cycles. Reactions were performed in triplicate, and independent experiments were repeated three times. The RT-PCR data were analyzed using StepOne Software 2.1, and primers are presented in **Table 1**.

**Table 1:** Sequences for primers.

Items	Direction	Sequence
IL6 primer	sense	ACTCACCTCTTCAGAACGAATTG
	reverse	CCATCTTTGGAAGGTTCAGGTTG
IL8 primer	sense	ACTCCAAACCTTTCCACCCC
	reverse	TTCTCAGCCCTCTTCAAAAACTTC
GAPDH primer	forward	GGTCACCAGGGCTGCTTTTA
	reverse	GGATCTCGCTCCTGGAAGATG
ICAM1 primer	forward	ACGTTGGATGAGCACTCAAGGGGAGGTCAC
	reverse	ACGTTGGATGGCTACCACAGTGATGATGAC

### Enzyme-linked immunosorbent assay

The levels of soluble IL-6/8/TNFα in the supernatant of primary MTCAFs and the supernatant were measured using an enzyme-linked immunosorbent assay (ELISA) kit (Jiangsu Meimian industrial Co., Ltd., TNFα: MM-0132M1, IL-6: MM-0163M1, and IL-8:MM-0123M1), according to the manufacturer’s instructions. The absorbance (450 nm) of each sample was detected on a standard automatic microplate reader (BioTek, USA).

### Cell invasion and migration assay

Colorectal cancer cell migration and invasion assay was conducted using 24-well Matrigel-coated Transwell inserts (BD Biosciences, San Diego, CA, USA). Approximately 2 × 10^5^ cells were seeded in serum-free medium in the upper chamber. Next, DMEM with FBS was added to the lower chamber, and after incubation at 37°C with 5% CO_2_ for 24–48 h, the non-filtered cells were removed using a cotton swab and the migratory cells were stained with 0.1% crystal violet solution. The invasive cells attached to the bottom surface of the filter were quantified under a light microscope (200×). The data are presented as the average number of cells from randomly chosen fields. Each treatment condition was assayed using triplicate filters, and all filters were counted in five areas.

### Wound healing

Using marker pen is to marker the 6-well plate with the ruler, which draw horizontal lines evenly (0.5-1cm). Each hole have to pass through at least 3 lines. Cell density is about 5*105 cells/pole. Next day, holding the head of the spear against the ruler and trying to keep it to the horizontal line in order to scratch. Wash the cells three times with PBS, remove the suspending cells, and add serum-free medium. Putting it into an incubator at 37°C with 5%CO2. Sampling at different hours and taking photos.

### Patients and samples

This is a retrospective cohort study. Colorectal cancers were obtained with informed consent from patients in Peking Union Medical College Hospital (Beijing, China) during January 2014 to December 2016. All specimens were collected using the protocols approved by the Ethics Committee of Peking Union Medical College Hospital. All patients were R0 resected and pathologically diagnosed with CRC.

### Immunohistochemistry

The resected tissue samples were fixed with formaldehyde, embedded in paraffin, and prepared into 4-m-thick sections. The slides were then dewaxed and hydrated. Next, we decreased the peroxidase activity by treating with 3% H_2_O_2_. The sections were blocked by using 10% normal goat serum and incubated with appropriate primary antibody overnight at 4°C. Then, PBS diluted secondary antibody at 1:100 was added followed by incubation at room temperature for 2 h. All immunostained sections were then lightly restained with hematoxylin. The results of immunohistochemistry (IHC) were evaluated by two pathologists independently. If the results were inconsistent, the final result would be judged by the third pathologist. The membrane staining of cells >5% was defined as ICAM-1 positive.

### Agilent expression profiling gene chip

The total RNA of the sample was quantified by NanoDrop ND-2000 (Thermo Scientific), and then, the RNA integrity was checked by Agilent Bioanalyzer 2100 (Agilent Technologies). After passing the RNA quality inspection, the labeling of the sample, the hybridization of the chip, and the elution refer to the standard process of the chip. First, total RNA is reverse-transcribed into double-stranded cDNA and then cRNA labeled with Cyanine-3-CTP (Cy3) is synthesized. The labeled cRNA is hybridized with the chip, and the original image is obtained by scanning with Agilent Scanner G2505C (Agilent Technologies) after elution.

### Animal experiments

All mice were maintained and manipulated according to the guidelines established by the Medical Research Animal Ethics Committee of Peking Union Medical University. The samples were randomly assigned. A mixture of 5 × 10^6^ HCT116 cells were re-suspended with 1 × 10^6^ cells or PBS (5:1) in 100 μl of PBS and subcutaneously injected into 6-week-old female athymic nude mice (BALB/C). Tumor formation was examined after 7 days. We detected the tumor size every three days, recorded the data, and finally calculated the volume (1/2 *the long side*the short side squared). When tumor volume reached 1–1.5 cm, the animals were sacrificed. Tissues were collected and sectioned followed by some sections being fixed with 10% buffered formalin for IHC analysis, whereas the others were preserved at −80°C for Western blotting.

### Writing statement

Participants have provided written informed consent to take part in the study.

### Statistical analysis

All data are expressed as means ± SD from at least three independent experiments. The statistics were analyzed by SPSS 25.0 statistical software (IBM, Armonk, USA). The relationship between the expression of ICAM-1 and disease-free survival (DFS) was evaluated by the Kaplan–Meier method. DFS was defined as the time from complete resection of tumor to disease recurrence. Statistical analysis was performed using two-tailed t-tests and one-way ANOVA. P < 0.05 was considered statistically significant. Each experiment was repeated at least three times to obtain a P-value and to control for systematic errors.

## Results

### Exosomes derived from HCT116 (H-Exos) induce the differentiation of MSCs into MTCAFs

**Figure 1:**
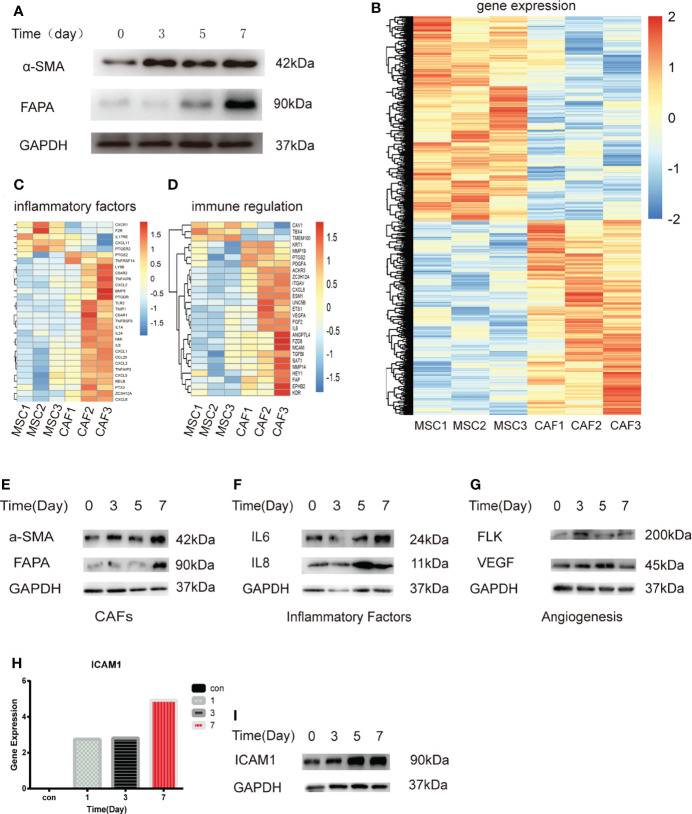
Transcriptome analysis of MSCs treated with H-Exos. **(A)** Detection of genes associated with MTCAF using Western blotting with the situation of MSCs with H-Exos at days 0, 3, 5, and 7. **(B)** Heat map showing the differentially expressed genes (DEGs) in HCT116-exos–treated MSCs (CAF1-3) and control MSCs (MSC1-3). **(C)** DEGs associated with inflammatory factors. **(D)** DEGs associated with immune regulation. **(E)** Detection of genes associated with MTCAF using Western blotting with the situation of co-culturing with HCT116 cells and MSCs at days 0, 3, 5, and 7. **(F)** Detection of genes associated with inflammatory factors using Western blotting. **(G)** Detection of genes associated with angiogenesis using Western blotting. **(H)** The expression of ICAM-1 was measured at days 0, 1, 3, and 7 by expression profiling gene chip **(I)** The expression of ICAM-1 using Western blotting at days 0, 1, 3, and 7.

Previous studies have shown that MSCs can differentiate into CAFs (4, 29). First, we applied H-Exos to induce MSCs differentiate into CAFs, which is named MTCAFs, and we found that MTCAFs have the higher CAF-specific gene expression (α-SMA and FAPA) (**Figure 1A**). The characteristics of H-Exos are presented in **Supplementary Figure 1**. Next, we evaluated the transcriptomic alterations and identified activated proteins in MTCAFs compared with MSCs; MTCAFs was kept into a transcriptionally active state, which was demonstrated by an increased number of upregulated genes (**Figure 1B**). Meanwhile, clustering identified upregulation of gene markers related to cell secreted inflammatory factors and immune regulation in MTCAFs compared with MSCs (**Figures 1C, D**). To be similar to the physiologic al conditions, we applied the co-culturing system, and we found that the co-culturing effect with HCT116 cells and MSCs is the same as that in exosomes secreted from HCT116 cells with MSCs (**Figure 1E**). Western blotting analysis showed an increase in the inflammatory and angiogenesis proteins from transcriptome analysis results (**Figures 1F, G**). In addition, the Agilent expression profiling gene chip results showed that ICAM-1 expression increased gradually during MSCs differentiation, and Western blotting analysis showed the same effect (**Figures 1H, I**). In conclusion, HCT116 cells can promote the differentiation of MSCs into MTCAFs and screen key gene ICAM-1 during the differentiation process.

### ICAM-1 might act as an indication for the progression of clinical colon cancer

**Figure 2:**
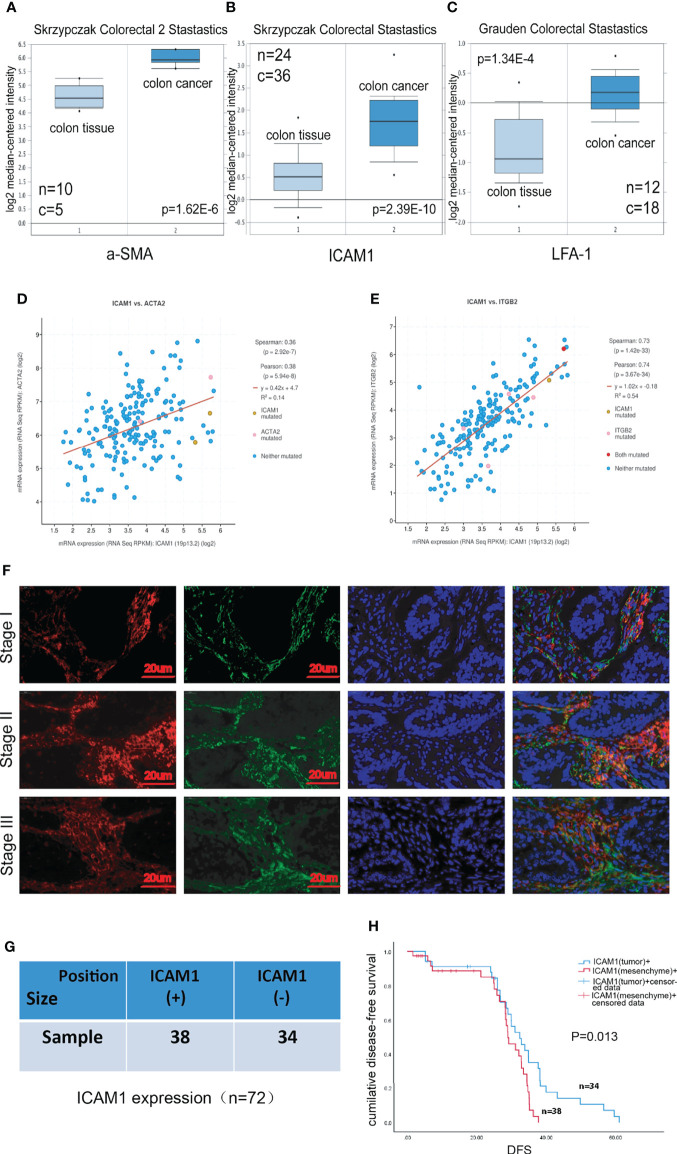
ICAM-1 expression has a poor prognosis in patients. **(A–C)** The expression analysis in relation to ICAM-1, α-SMA, and LFA-1 from Oncomine Database, respectively. **(D, E)** cBioPortal Database indicated the correlation between ICAM-1 and α-SMA or LFA-1. **(F)** Colon cancer tissues specimens consisting of patients with stage I, II, and III CRC were immunofluorescent staining with antibody against ICAM-1 (red), α-SMA(green), and nucleus (blue). **(G)** The enrolled 72 patients were divided into two groups including only mesenchymal ICAM-1 positive (30) and mesenchymal ICAM1 negative (31). **(H)** Kaplan–Meier curves for DFS of mesenchymal ICAM-1 expression. *p < 0.05, **p < 0.01, and ***p < 0.001.

To explore the correlations between ICAM-1 expression and progression and prognosis of patients with colon cancer, we collected patients samples with colon cancer from Oncomine Database, which includes paracarcinoma tissue and colorectal cancer tissue. The Oncomine analysis showed that α-SMA, ICAM-1, and LFA-1 exhibited a higher expression in colorectal cancer compared with colon tissue (**Figures 2A–C**), and ICAM-1 was positively correlated with α-SMA and LFA-1 (**Figures 2D, E**). To further clarify the function of these genes, we used clinical specimens for further analysis. The expression of ICAM-1 and α-SMA in tumor tissue of patients with stage I, II, and III CRC were obtained by using immunofluorescent staining. The result showed that ICAM-1 and α-SMA were co-expressed in clinical samples (**Figure 2F**). Next, we enrolled 72 patients (n = 72), 38 samples showed ICAM-1 positive, and 34 samples showed ICAM-1 negative (**Figure 2G**). On basis of this, we analyze the relationship between ICAM-1 expression and patient survival, and the result showed that the DFS of patients with positive ICAM-1 expression was significantly shorter than that of ICAM-1–negative patients [(28.06 ± 1.47) months *vs*. (38.87 ± 3.35) months, P = 0.013] (**Figure 2H**). These results suggest that ICAM-1 is inversely associated with survival in patients with colorectal cancer.

### ICAM-1 is critical for the migration and homing abilities of MTCAFs

**Figure 3:**
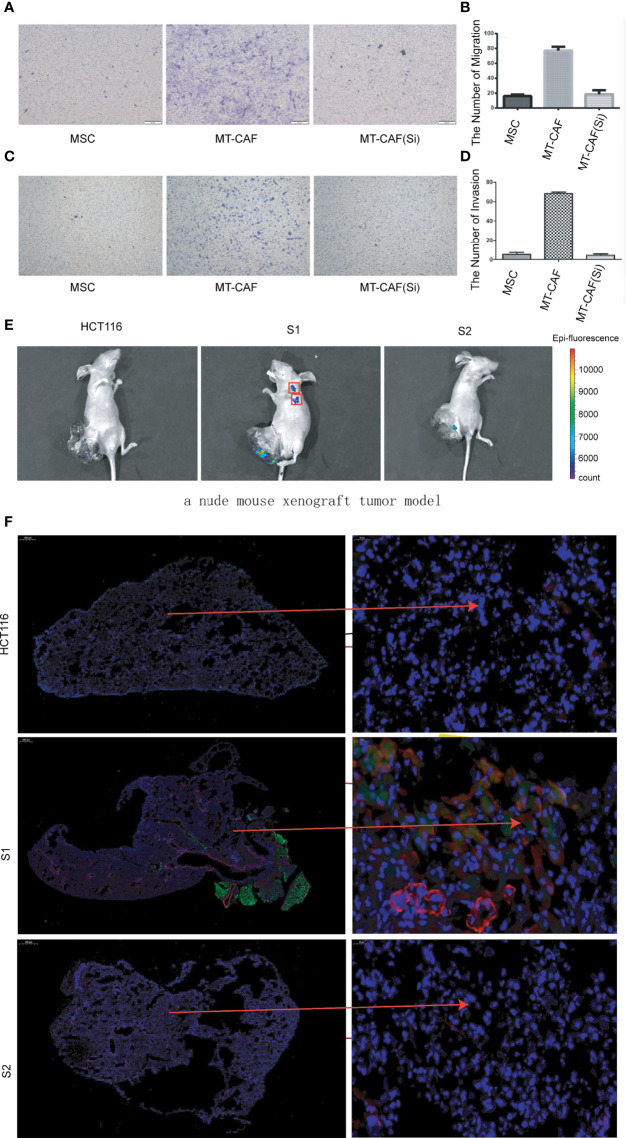
Knockdown ICAM-1 in MTCAFs attenuates their migration and homing abilities. **(A, B)** Transwell migration assays to evaluate the MTCAF migratory capacity were performed and are represented. The left side shows a representative microscopic image of the crystal violet staining. The right shows the quantitative results. **(C, D)** Transwell migration assays to evaluate the MTCAF invasion capacity were also performed and are represented. The left side shows a representative microscopic image of the crystal violet staining. The right shows the quantitative results. **(E)** *In vivo* fluorescence image showing the effect of combined MSC transplantation on tumor metastasis. Three groups of mice were transplanted with HCT-116 5 × 10^6^ cells, HCT-116 5 × 10^6^ cells + MSC 1 × 10^6^ cells, and HCT-116 5 × 10^6^ cells + MSC with ICAM1 knocking down 1 × 10^6^ cells. The MSC cell lines carried GFP. **(F)** MTCAF density was measured using immunofluorescence staining in mice lung tissues by staining α-SMA and GFP transfected in MSCs.

To further validate the function of ICAM-1 in the process of MSC differentiating into MTCAFs, we detected the invasion and migration abilities of MTCAFs, and the results showed that MTCAFs with ICAM-1 knockdown presented with significantly decreased abilities of migration (**Figures 3A, B**) and invasion (**Figures 3C, D**) compared with MTCAFs, which means that the ICAM-1 may play an important role in the MTCAFs. To further understand the situation of MTCAF homing, we built a nude mouse xenograft tumor model (mice, n = 10). HCT116 cells were subcutaneously co-implanted with MSCs (S1), MSCs with ICAM-1 knockdown (S2) and PBS (NC) at a ratio of 5:1. We next detected the abilities of distant migration by *in vivo* fluorescence image, and we found that homing to the lungs in S1 group was more stronger than that in S2 group (**Figure 3E**). To determine which cells migrate to the lungs, we next detected the CAF-specific marker genes (α-SMA and FAPA) by immunofluorescence staining, and the results showed that MTCAFs migrate to the lung in the S1 group compared with the other two groups (**Figure 3F**). These results show that the ICAM-1 gene mediates the movement of MTCAFs, which may influence the progression of colon cancer cells.

### ICAM-1 regulates the inflammatory secretion of MTCAFs and mediates the inflammatory microenvironment

**Figure 4:**
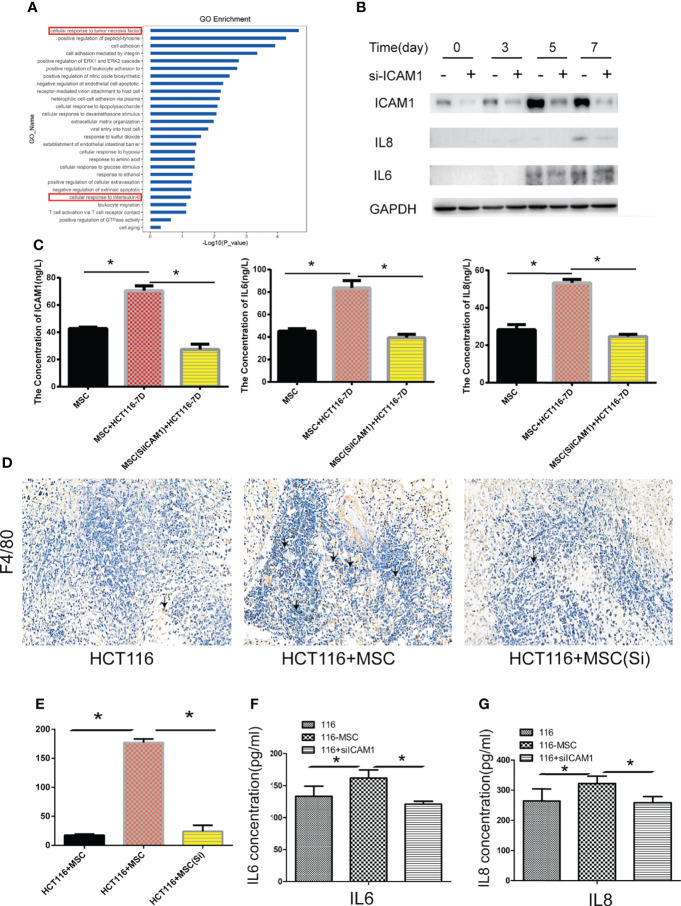
ICAM-1 mediates the inflammatory microenvironment. **(A)** Go enrichment analysis showed that inflammatory signaling pathways in MTCAF were significantly activated with the situation of H-Exos stimulation. **(B)** The expression of IL6 and IL8 was detected by Western blotting (WB) in MTCAFs or MTCAFs with knocking down ICAM1 at days 0, 3, 5, and 7. **(C)** ELISA detection detected the ICAM1, IL6, and IL8 expression from cellular supernatant with ICAM1 or without ICAM1. **(D, E)** Macrophage infiltration into tumor tissues was examined using immunohistochemistry for the detection of the F4/80. Representative images of F4/80 stainings for each group are shown (magnification, × 400). Panel **(E)** shows the quantitative results. **(F, G)** IL-6 and IL-8 from mice serum levels were evaluated using ELISA in the three different groups (n = 5). *p < 0.05, **p < 0.01, and ***p < 0.001.

In our study, the transcriptomic analysis indicated that H-Exos activated different signals including TNFα and IL6 signaling pathways in MTCAFs (**Figure 4A**). Among the inflammatory factors, Western blotting analysis revealed that the expression of IL-6 and IL-8 was decreased, whereas MTCAFs were knocked down by ICAM-1 (**Figure 4B**). This result was further verified by ELISA to detect ICAM-1, IL-6, and IL-8 concentration of serum on day 7 (**Figure 4C**). In addition, by accessing immune cell infiltration *in vivo*, we showed that the number of F4/80 macrophages was lower in tumors in S2 group compared with S1 group (**Figures 4D, E**). Some studies suggest that the inflammatory factors, IL-6, IL-8, and TNFα, are major regulators of tumor stroma interaction in the cancer microenvironment (32–35). We examined their expression levels in mice serum by ELISA (n = 5) and found increased levels of IL-6 and IL-8 (**Figures 4F, G**). In conclusion, MTCAFs with ICAM-1 are able to mediates the inflammatory microenvironment.

### MTCAFs regulate colon cancer cell invasion and migration *via* secreting ICAM-1

**Figure 5:**
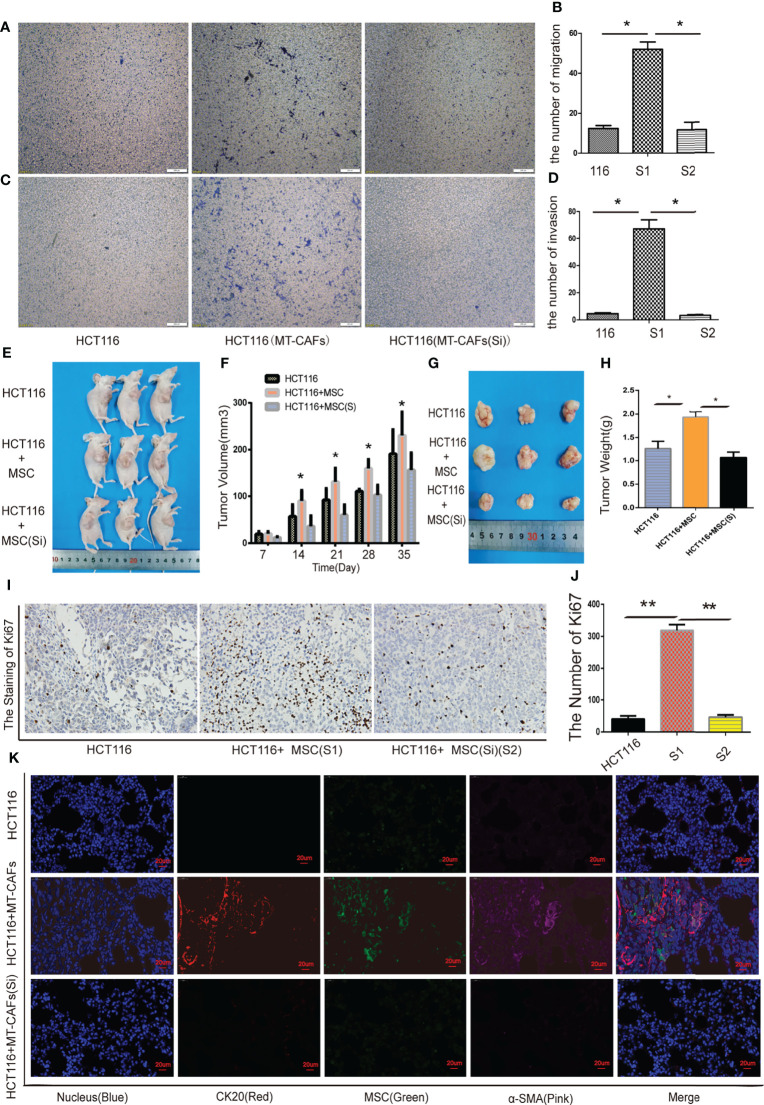
ICAM-1 from MTCAFs influences the progression of colon cancer cells. **(A, B)** Transwell migration assays to evaluate the HCT116 cells migration capacity were also performed. Panel **(A)** indicates a representative microscopic image of the crystal violet staining. Panel **(B)** shows the quantitative results. **(C, D)** Transwell assays to evaluate the HCT116 cells invasion capacity were also performed. Panel **(C)** indicates a representative microscopic image of the crystal violet staining. Panel **(D)** shows the quantitative results. **(E)** Representative photographs of HCT116 tumors generated in nude mice eco-implanted with MSCs (S1), MSCs after ICAM-1 silencing (S2), or PBS (NC) at a ratio of 5:1. **(F)** The quantitative data referent to **(E)**. **(G)** The weight (g) of tumors were discorded. **(H)** The quantitative data referent to **(G)**. **(I, J)** The expression of Ki67 in tumor tissue of mice in S1, S2, and NC groups was detected by immunohistochemistry. **(J)** The quantitative data. **(K)** MSCs and HCT116 density were measured using immunofluorescence staining in mice lung tissue (CK20, red, represents colorectal cancer cells; α-SMA, pink, represents MTCAFs; green, fluorescent protein carried by MSCs; and Hoechst3342, blue, represents the nucleus. *p < 0.05, **p < 0.01, and ***p < 0.001.

In our study, we further explored the effect of MTCAF-derived ICAM-1 on the HCT116 cells, and we found that migration abilities (**Figures 5A, B**) and invasion abilities (**Figures 5C, D**) of HCT116 cells were obviously weakened at day 7 when MTCAFs were knocked down. We next analyzed whether MTCAFs with ICAM-1 knockdown affected the tumor progression and immune environment by using a nude mouse xenograft tumor model (mice, n = 10). HCT116 cells were subcutaneously co-implanted with MSCs (S1), ICAM-1 knockdown MSCs (S2), or PBS (NC) at a ratio of 5:1. Mice in S1 group promoted the growth of tumor compared with the other two groups *in vivo* (**Figures 5E, F**). Next, we assessed the tumor weight by excising the tumor from mice, and the results were similar to those observed for tumor growth (**Figures 5G, H**). Meanwhile, Ki67 staining was performed, and the results showed that the proliferation capacity of the S1 group was significantly higher than NC group, whereas knocking down ICAM-1 significantly decreased the ability of proliferation (**Figures 5I, J**). We found that HCT116 cells can also migrate into the lungs in S1 group but not in S2 group and NC group (**Figure 5K**). These results show that ICAM-1 mediates progression of colon cancer cells.

### ICAM-1 secreted from MTCAFs mediates the STAT3 and AKT signaling pathway in colon cancer cells

**Figure 6:**
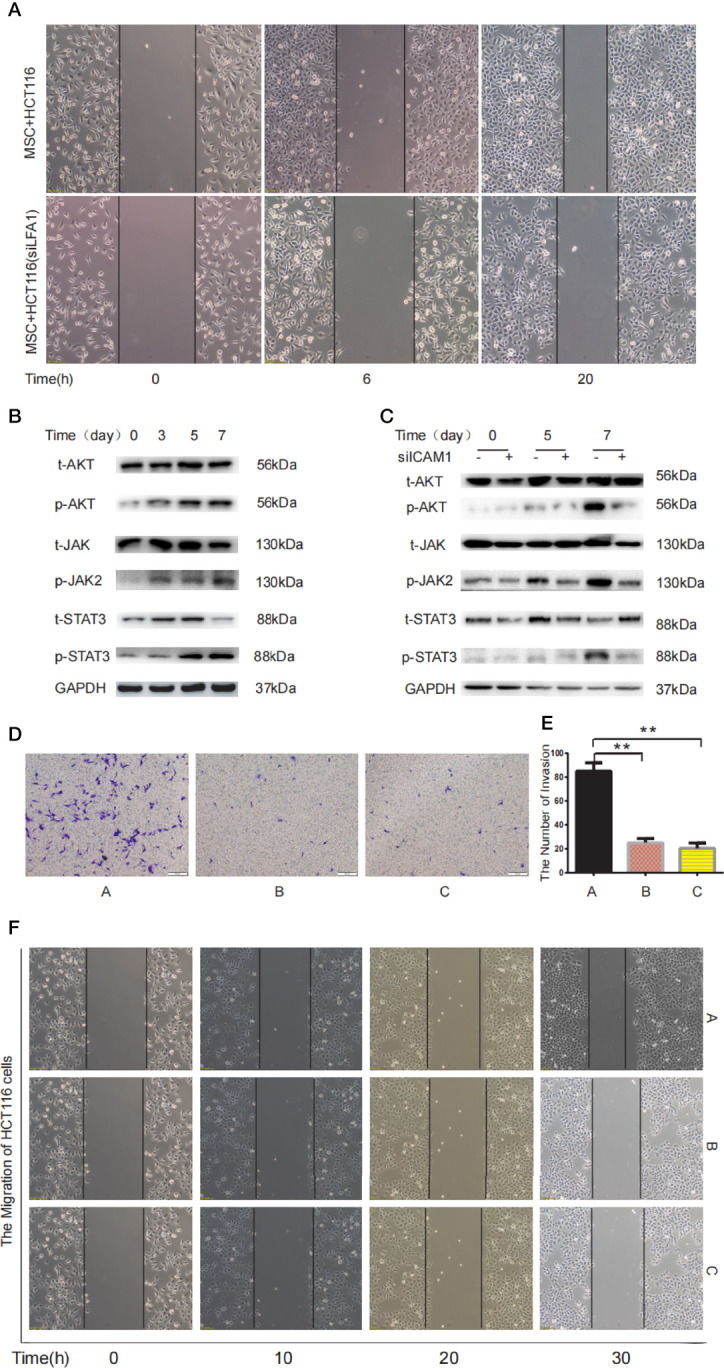
MTCAF-derived ICAM-1 mediates STAT3 and Akt signaling pathways in colon cancer cells. **(A)** The migration ability of HCT116 cells was detected by wound healing in two different treatment groups including MSC co-culturing with HCT116 cells or HCT116 cells with LFA inhibitor (44 nM) groups. **(B)** Western blotting assay was used to detect the phosphorylation of AKT and STAT3 in HCT116 cells when HCT116 cells and MSCs were co-cultured at days 0, 3, 5, and 7. GAPDH was used as the control group. **(C)** Western blotting was used to detect AKT and STAT3 signaling pathway in HCT116 cells with co-culturing with MSCs or MSCs knocking down ICAM-1. GAPDH was used as the control group. **(D)** Invasive ability of HCT116 cells was measured using the Transwell assay. The left shows the microscopic image of the crystal violet staining [group A represents the HCT116 and MSC co-culture group, group B represents the HCT116 and MSC co-culture group with AKT inhibitor (10 10 μM) group, and group C represents the HCT116 and MSC co-culture group with STAT3 inhibitor (2.14 μM) group). **(E)** The microscopic image of the crystal violet staining. **(F)** The wound healing was used to detect migration ability of HCT116 cells at different time (group A represents the HCT116 and MSC co-culture group, group B represents the HCT116 and MSC co-culture group with AKT inhibitor group, and group C represents the HCT116 and MSC co-culture group with STAT3 inhibitor group). **p < 0.01 and ***p < 0.001.

LFA-1 has been reported to be the most important ICAM-1 receptor (31). In our study, wound healing assay confirmed that ICAM-1 secreted by MTCAFs regulates migration of HCT116 cells by interacting with LFA-1 expressed on HCT116 cells (**Figure 6A**). Western blotting results showed that JAK, STAT3, and AKT were also activated in HCT116 cells co-cultured with MTCAFs after day 3 (**Figure 6B**), whereas MTCAFs were knocked down by ICAM-1, and the phosphorylation of JAK, STAT3, and AKT in HCT116 cells was significantly decreased (**Figure 6C**). Next, we detected the migration and invasion ability of HCT116 cells in different treatment groups, including HCT116 and MSC co-culture group (group A), HCT116 and MSC co-culture plus STAT3 inhibitor group (group B), and HCT116 and MSC co-culture plus AKT inhibitor group (group C). Transwell results showed that HCT116 cell invasion was significantly reduced in groups B and C compared with group A (**Figures 6D, E**). Wound healing test results showed that the migration ability of HCT116 cells in groups B and C was significantly weakened compared with group A (**Figure 6F**). Finally, immunohistochemistry demonstrated that MTCAFs can activate AKT and STAT3 signaling pathways in tumor tissues in nude xenograft tumor models (**Supplementary Figure 2**). These results suggest that MTCAF-derived ICAM-1 promotes the progression of colon cancer cells by binding LFA-1 to activate STAT3 and AKT signaling pathways.

## Discussion

It has been well established that CAFs promotes various tumor progression, and CAFs originate in a variety of cells including MSCs, endotheliocyte, and epithelial cell (36). In our study, we mainly use MSC-derived CAFs (MTCAFs).

Mechanistically, this mainly contributed to the matrix deposition and remodeling, interactions with cancer cells *via* extensive reciprocal signaling, and crosstalk with infiltrating immune cells (30, 37–39). Although recent studies have found that CAFs attribute to the progression of colon cancer (40, 41), the origin and role of CAFs in colon cancer and its mechanism have needed to be fully elucidated. Here, we confirm that MSC-derived CAFs promote the growth, migration, and invasion of colon cancer cells and testify the critical role of CAFs in the microenvironment of colon cancer.

Recent reports have shown that CAFs can secrete various cytokines such as growth factor, inflammatory factors, and chemokine, which can stimulate diverse signaling pathways and biological functions of different cancers (10). Previous studies have shown that several cytokines in CAFs were increased, which contribute to the progression of various cancer including IL8, IL6, and TGF-β1 (10, 42–44). In our work, we identified a novel cytokine, ICAM-1, which is a transmembrane molecule stabilizing cell–cell and cell-extracellular matrix interactions and facilitating transendothelial transmigration (45, 46). MTCAF-derived ICAM-1 has double roles: It not only regulates the growth and migration of MTCAFs by mediating the expression of IL6 and IL8 in MTCAFs but also can promote the proliferation and invasion of cancer cells. Previous study has shown that the prognosis of the patients with ICAM-1–negative tumors was significantly poorer than that of those with ICAM-1–positive tumors (47). This result is not contradictory with our result. We consider that this difference between our results and the previous reports may be due to ICAM-1 derived from MTCAFs. Our results further verified this hypothesis. We found that ICAM-1 expression was high in fibroblasts (i.e., mesenchymal cells of tumor tissue) of tumor tissue in patients with different clinical stages, which negatively correlated with patient survival, consistent with our results *in vivo* and *in vitro*.

One of the critical problems is the molecular mechanism of ICAM-1 derived from MTCAFs action on colon cancer cells in our study. It has been found that CAFs can be activated by some cytokines in TME, such as IL6, IL8, and Fibroblast growth factor 2 (FGF2) (43, 48), which further activates various pathways including IL6-STAT3 and AKT signaling pathways (43, 49). Interestingly, these pathways have been also shown to regulate ICAM-1 expression (20). Therefore, we found that MTCAF-derived ICAM-1 promotes the progression by activating the STAT3 and AKT signaling in colon cancer cells. However, this specific question needs further study.

Most studies have demonstrated that ICAM-1 regulates cancer metastasis *via* the binding receptor, LFA-1, which can activate numerous pathways (50–52). ICAM‐1–induced tumor COX‐2 impaired the antitumor activity *via* binding LFA-1 during hepatic metastasis (52). The expression of inflammatory cytokines, such as IL-1β, TNFα, IL-6, and IFN-γ, tightly regulates ICAM-1 expression (53, 54). In addition, the ICAM-1/LFA-1 pathway regulates important cell–cell interactions including leukocyte adhesion and migration, especially the killing of tumor cells by natural killer cells and cytotoxic T lymphocytes (CTLs) (55, 56). At present, various tumor cells have been shown to highly express ICAM-1 that is known to be a potent ligand for LFA-1 on CTLs. Most studies have revealed that ICAM-1 plays an important role in the progress and metastasis of many cancers (21, 57–59). However, the function of ICAM-1 in CAFs has not been revealed in the TME of colorectal cancer. In our study, we found that ICAM-1 derived from MTCAFs promotes the migration and invasion of colorectal cancer cells by binding LFA-1 receptor of colon cancer, subsequently activating AKT and STAT3 in HCT116 cells. The possible mechanism is that MTCAFs activate the AKT and STAT3 signaling pathways in colon cancer cells *via* the ICAM-1/LFA-1axis.

Although we confirmed the important role of MTCAF-derived ICAM-1 in colorectal cancer, there are still many limitations, such as stage IV patients were not included in our study. One reason for lacking of stage IV patients was that those patients rarely underwent surgery in the past years. We can enroll larger sample capacity to further explore the correlation between ICAM-1 and clinical features in this should be the future.

## Conclusion

In summary, we found that ICAM-1 secreted from CAFs enhances the migration and invasion ability of colorectal cancer cells by activating the AKT and STAT3 pathway in cancer cells (**Figure 6**). Our results provide a better cognition of how CAFs work in the TME in colorectal cancer. MTCAF-derived ICAM-1 may play an important role in promoting cancer metastasis and can serve as a predictive and prognostic biomarker in colorectal cancer.

## Data availability statement

The datasets presented in this study can be found in online repositories. The names of the repository/repositories and accession number(s) can be found in the article/**Supplementary Material**.

## Ethics statement

The studies involving human participants were reviewed and approved by This is a retrospective cohort study. Colorectal cancers were obtained with informed consent from patients in Peking Union Medical College Hospital (Beijing, China) during January 2014 to December 2016. All specimens were collected using the protocols approved by the Ethics Committee of Peking Union Medical College Hospital. All patients were R0 resected and pathologically diagnosed with CRC. The patients/participants provided their written informed consent to participate in this study.

The animal study was reviewed and approved by the Ethics Committee at the Chinese Academy of Medical Sciences and Peking Union Medical College.

## Author contributions

The study was designed by QH, ZS, CB, and RZ. CX carried out the experiments, performed the statistical analyses, and wrote the manuscript. YG and XL contributed to the statistical analyses. MZ and YY helped do some experiments. All authors have read and approved the final manuscript. All authors contributed to the article and approved the submitted version.

## Funding

This work was supported by the National Key Research and Development Program of China (2016YFA0101000, 2016YFA0101003, and 2018YFA0109800), CAMS Innovation Fund for Medical Sciences (2017-I2M-3-007), the 111 Project (B18007), and National Natural Science Foundation of China (81672313 and 81700782).

## Conflict of interest

The authors declare that the research was conducted in the absence of any commercial or financial relationships that could be construed as a potential conflict of interest.

## Publisher’s note

All claims expressed in this article are solely those of the authors and do not necessarily represent those of their affiliated organizations, or those of the publisher, the editors and the reviewers. Any product that may be evaluated in this article, or claim that may be made by its manufacturer, is not guaranteed or endorsed by the publisher.

## Supplementary material

Supplementary Figure 1 Characterisation of exosomes derived from HCT116 cells (HCT116-exos) and *in vitro* uptake assay results. **(A)** The morphology of HCT116-exos was assessed using electron microscopy. **(B)** HSP70, HSP90, and CD63 expression in HCT116cells and HCT116-exos was analyzed using Western blotting. **(C)** HCT116-exos size distribution was evaluated by NTA analysis. **(D)** Uptake of DiR-labelled HCT116-exos by MSCs was also evaluated after 10h.

Supplementary Figure 2 Knocking down ICAM-1 from MTCAFs attenuates STAT3 and AKT signaling *in vivo*. (E) (A/B)AKT and STAT3 signaling pathways were measured using immunohistochemical staining in mice tumor tissues.

## Abbreviations

MSCs, mesenchymal stem cells; hADSCs, human adipose-derived mesenchymal stem cells; CAFs, cancer-associated fibroblasts; MTCAF, MSC-transformed CAF; α-SMA, alpha–smooth muscle actin; FAPA, fibroblast activation protein alpha; CRC, colorectal cancer; TME, tumor microenvironment; IL-6, interleukin-6; CCL2, CC-chemokine ligand 2; SDF-1/CXCL-12, stromal cell-derived factor 1; ICAM-1, Intercellular adhesion molecule-1; LFA-1, leukocyte-function associated antigen-1; DMEM, Dulbecco’s Modified Eagle Medium; TEM, transmission electron microscopy; IHC, immunohistochemistry; IF, immunofluorescence.
