# Supplementary figures and images for: Mesenchymal stem cells derived from adipose tissue accelerate the progression of colon cancer by inducing a MTCAF phenotype via ICAM1/STAT3/AKT axis
Source: Front Oncol. 2022 Aug 9;12:837781. doi: 10.3389/fonc.2022.837781 (PMC9398219; doi:10.3389/fonc.2022.837781)

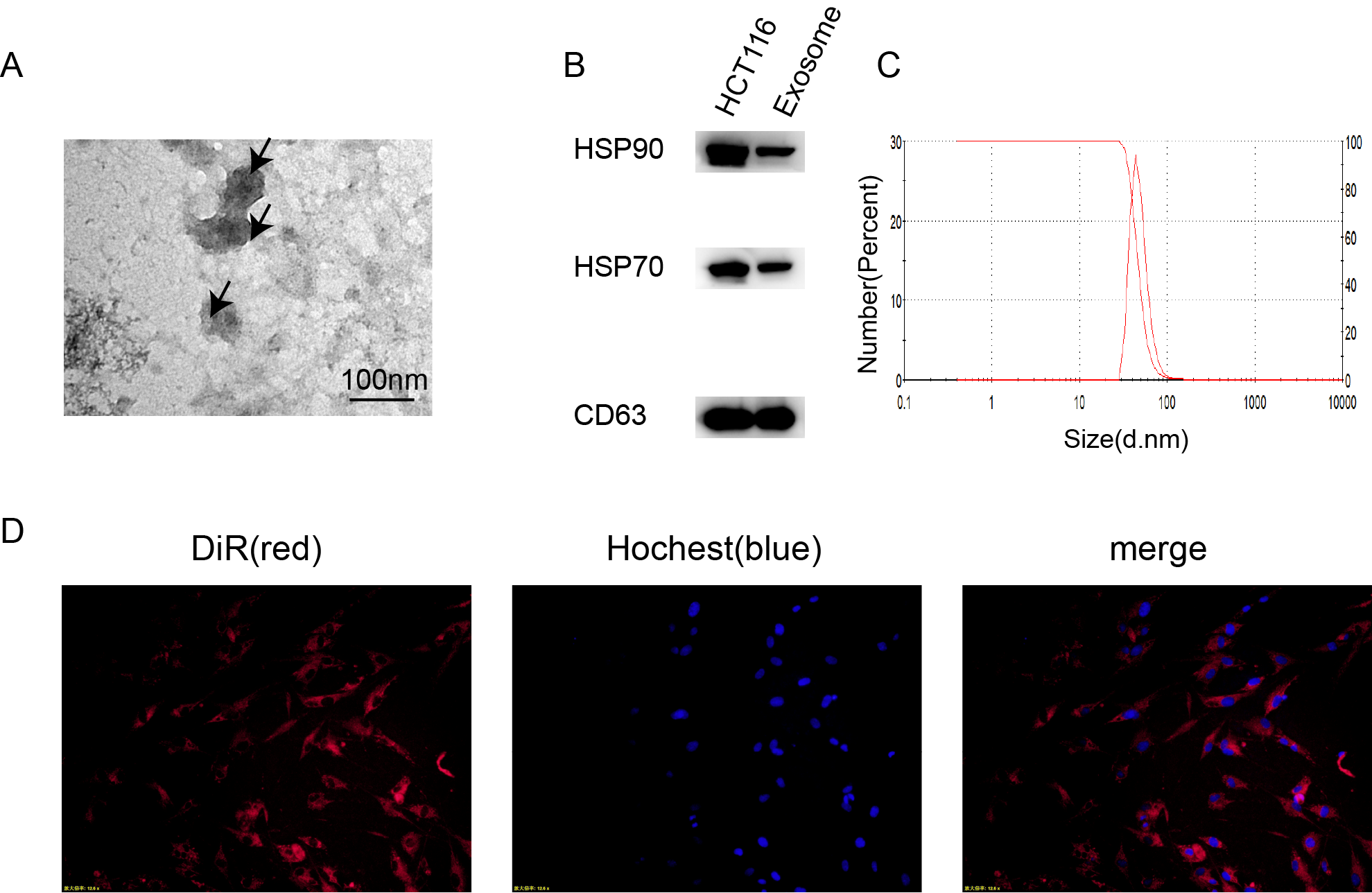

Supplement: Supplementary Figure 1 — Characterisation of exosomes derived from HCT116 cells (HCT116-exos) and in vitro uptake assay results. (A) The morphology of HCT116-exos was assessed using electron microscopy. (B) HSP70, HSP90, and CD63 expression in HCT116cells and HCT116-exos was analyzed using Western blotting. (C) HCT116-exos size distribution was evaluated by NTA analysis. (D) Uptake of DiR-labelled HCT116-exos by MSCs was also evaluated after 10h. [file Image_1.tif]

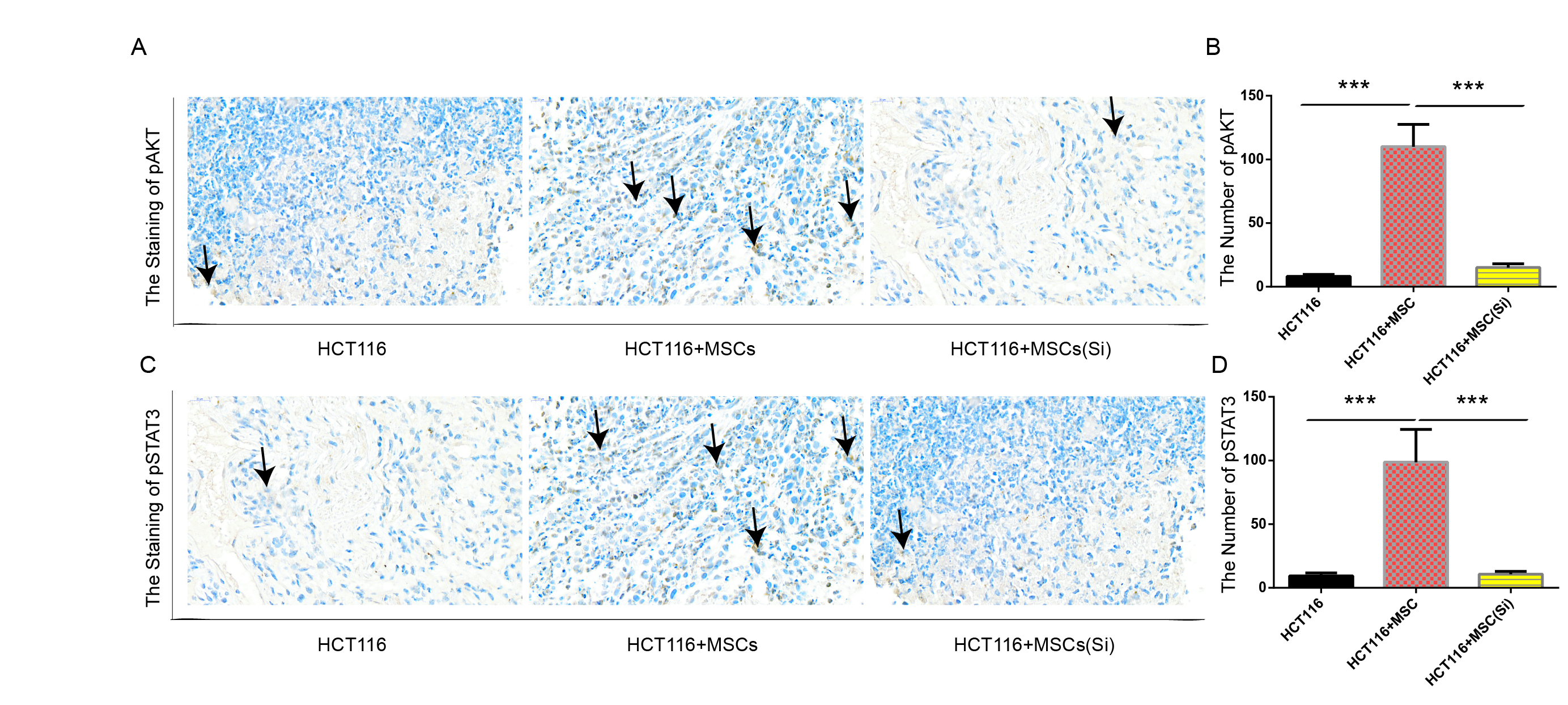

Supplement: Supplementary Figure 2 — Knocking down ICAM-1 from MTCAFs attenuates STAT3 and AKT signaling in vivo. (E) (A/B)AKT and STAT3 signaling pathways were measured using immunohistochemical staining in mice tumor tissues. [file Image_2.tif]
